# (*E*)-4-Chloro-*N*-(2,4,6-trimethyl­benzyl­idene)aniline

**DOI:** 10.1107/S1600536811027024

**Published:** 2011-07-09

**Authors:** Ying Guo, Meng-Xin Pan, Hai Xiang, Wen-Hong Liu, Zhong-Cheng Song

**Affiliations:** a College of Bioengineering, Zhejiang Chinese Medical University, Hangzhou 310053, People’s Republic of China

## Abstract

In the title compound, C_16_H_16_ClN, the dihedral angle between the benzene rings is 24.61 (13)°. In the crystal, only van der Waals inter­actions occur between neighbouring mol­ecules.

## Related literature

For related structures, see: Nie (2008 ▶); Cui *et al.* (2009 ▶); Sun *et al.* (2011*a* ▶,*b* ▶).
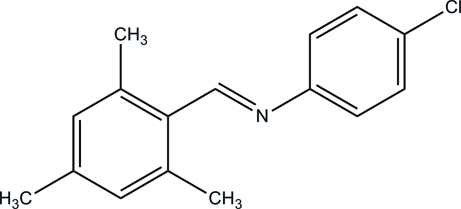


## Experimental

### 

#### Crystal data

C_16_H_16_ClN
*M* *_r_* = 257.75
Monoclinic, 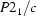

*a* = 7.198 (2) Å
*b* = 12.398 (4) Å
*c* = 15.865 (5) Å
β = 102.296 (4)°
*V* = 1383.3 (7) Å^3^
*Z* = 4
Mo *K*α radiation
μ = 0.26 mm^−1^
*T* = 293 K
0.31 × 0.30 × 0.29 mm

#### Data collection

Bruker APEXII CCD diffractometer
Absorption correction: multi-scan (*SADABS*; Bruker, 2004 ▶) *T*_min_ = 0.924, *T*_max_ = 0.929
7071 measured reflections
2553 independent reflections
1593 reflections with *I* > 2σ(*I*)
*R*_int_ = 0.042

#### Refinement

*R*[*F* ^2^ > 2σ(*F* ^2^)] = 0.046
*wR*(*F* ^2^) = 0.136
*S* = 1.04
2553 reflections
167 parameters
H-atom parameters constrained
Δρ_max_ = 0.19 e Å^−3^
Δρ_min_ = −0.16 e Å^−3^

### 

Data collection: *APEX2* (Bruker, 2004 ▶); cell refinement: *SAINT* (Bruker, 2004 ▶); data reduction: *SAINT*; program(s) used to solve structure: *SHELXS97* (Sheldrick, 2008 ▶); program(s) used to refine structure: *SHELXL97* (Sheldrick, 2008 ▶); molecular graphics: *SHELXTL* (Sheldrick, 2008 ▶); software used to prepare material for publication: *SHELXTL*.

## Supplementary Material

Crystal structure: contains datablock(s) global, I. DOI: 10.1107/S1600536811027024/hb5944sup1.cif

Structure factors: contains datablock(s) I. DOI: 10.1107/S1600536811027024/hb5944Isup2.hkl

Supplementary material file. DOI: 10.1107/S1600536811027024/hb5944Isup3.cml

Additional supplementary materials:  crystallographic information; 3D view; checkCIF report

## Figures and Tables

**Fig. 1.**
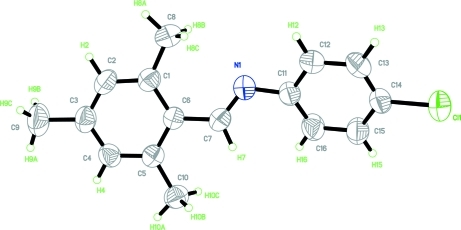
The molecular structure of the title compound with 50% probability displacement ellipsoids for non-hydrogen atoms.

## supplementary crystallographic information

### Comment

We report here the crystal structure of the title new Schiff base compound, (I). In (I) (Fig. 1), the geometric parameters of the title compound agree well with reported similar structures (Nie, 2008; Cui *et al.*, 2009; Sun *et al.*, 2011*a*,b). The dihedral angle between the two aromatic rings in the Schiff base molecule is 24.61 (13)°,

### Experimental

A mixture of 2,4,6-trimethylbenzaldehyde (5 mmol), 4-chloroaniline (5 mmol) and methanol (40 ml) was refluxed for 2 h. It was then allowed to cool and filtered. Recrystallization of the crude product from methanol yielded colorless blocks of (I).

### Refinement

H atoms were positioned geometrically and refined using the riding-model approximation, with C—H = 0.93–0.96 Å and *U*_iso_(H) = 1.2*U*_eq_(C) or 1.5U_eq_(methyl C).

### Crystal data

**Table:**

C_16_H_16_ClN	*F*(000) = 544
*M**_r_* = 257.75	*D*_x_ = 1.238 Mg m^−^^3^
Monoclinic, *P*2_1_/*c*	Mo *K*α radiation, λ = 0.71073 Å
Hall symbol: -P 2ybc	Cell parameters from 1950 reflections
*a* = 7.198 (2) Å	θ = 2.6–24.8°
*b* = 12.398 (4) Å	µ = 0.26 mm^−^^1^
*c* = 15.865 (5) Å	*T* = 293 K
β = 102.296 (4)°	Block, colorless
*V* = 1383.3 (7) Å^3^	0.31 × 0.30 × 0.29 mm
*Z* = 4	

### Data collection

**Table:**

Bruker APEXII CCD diffractometer	2553 independent reflections
Radiation source: fine-focus sealed tube	1593 reflections with *I* > 2σ(*I*)
graphite	*R*_int_ = 0.042
φ and ω scans	θ_max_ = 25.5°, θ_min_ = 2.6°
Absorption correction: multi-scan (*SADABS*; Bruker, 2004)	*h* = −8→8
*T*_min_ = 0.924, *T*_max_ = 0.929	*k* = −15→12
7071 measured reflections	*l* = −19→19

### Refinement

**Table:**

Refinement on *F*^2^	Secondary atom site location: difference Fourier map
Least-squares matrix: full	Hydrogen site location: inferred from neighbouring sites
*R*[*F*^2^ > 2σ(*F*^2^)] = 0.046	H-atom parameters constrained
*wR*(*F*^2^) = 0.136	*w* = 1/[σ^2^(*F*_o_^2^) + (0.0476*P*)^2^ + 0.5504*P*] where *P* = (*F*_o_^2^ + 2*F*_c_^2^)/3
*S* = 1.04	(Δ/σ)_max_ < 0.001
2553 reflections	Δρ_max_ = 0.19 e Å^−^^3^
167 parameters	Δρ_min_ = −0.16 e Å^−^^3^
0 restraints	Extinction correction: *SHELXL97* (Sheldrick, 2008), Fc^*^=kFc[1+0.001xFc^2^λ^3^/sin(2θ)]^-1/4^
Primary atom site location: structure-invariant direct methods	Extinction coefficient: 0.024 (3)

### Special details

**Table:**

Geometry. All e.s.d.'s (except the e.s.d. in the dihedral angle between two l.s. planes) are estimated using the full covariance matrix. The cell e.s.d.'s are taken into account individually in the estimation of e.s.d.'s in distances, angles and torsion angles; correlations between e.s.d.'s in cell parameters are only used when they are defined by crystal symmetry. An approximate (isotropic) treatment of cell e.s.d.'s is used for estimating e.s.d.'s involving l.s. planes.
Refinement. Refinement of *F*^2^ against ALL reflections. The weighted *R*-factor *wR* and goodness of fit *S* are based on *F*^2^, conventional *R*-factors *R* are based on *F*, with *F* set to zero for negative *F*^2^. The threshold expression of *F*^2^ > σ(*F*^2^) is used only for calculating *R*-factors(gt) *etc*. and is not relevant to the choice of reflections for refinement. *R*-factors based on *F*^2^ are statistically about twice as large as those based on *F*, and *R*- factors based on ALL data will be even larger.

### Fractional atomic coordinates and isotropic or equivalent isotropic displacement parameters (Å^2^)

**Table:**

	*x*	*y*	*z*	*U*_iso_*/*U*_eq_	
C1	0.2061 (3)	0.3177 (2)	−0.07427 (15)	0.0528 (6)	
C2	0.1376 (4)	0.3161 (2)	−0.16278 (16)	0.0607 (7)	
H2	0.1204	0.2497	−0.1906	0.073*	
C3	0.0937 (4)	0.4085 (2)	−0.21154 (16)	0.0608 (7)	
C4	0.1175 (3)	0.5063 (2)	−0.16925 (16)	0.0568 (7)	
H4	0.0864	0.5692	−0.2010	0.068*	
C5	0.1867 (3)	0.51398 (19)	−0.08065 (15)	0.0489 (6)	
C6	0.2323 (3)	0.41907 (19)	−0.03188 (14)	0.0451 (6)	
C7	0.3158 (3)	0.4292 (2)	0.06073 (15)	0.0504 (6)	
H7	0.3552	0.4976	0.0811	0.061*	
C8	0.2484 (5)	0.2115 (2)	−0.02813 (18)	0.0752 (8)	
H8A	0.2283	0.1538	−0.0695	0.113*	
H8B	0.1655	0.2022	0.0114	0.113*	
H8C	0.3782	0.2107	0.0031	0.113*	
C9	0.0207 (5)	0.4020 (3)	−0.30862 (17)	0.0902 (10)	
H9A	0.0000	0.4735	−0.3319	0.135*	
H9B	−0.0967	0.3626	−0.3210	0.135*	
H9C	0.1129	0.3657	−0.3342	0.135*	
C10	0.2072 (4)	0.6250 (2)	−0.04014 (17)	0.0625 (7)	
H10A	0.1588	0.6781	−0.0833	0.094*	
H10B	0.3390	0.6392	−0.0163	0.094*	
H10C	0.1367	0.6281	0.0048	0.094*	
C11	0.4309 (4)	0.3741 (2)	0.20165 (15)	0.0544 (6)	
C12	0.5466 (4)	0.2942 (2)	0.24460 (18)	0.0742 (9)	
H12	0.5584	0.2297	0.2162	0.089*	
C13	0.6456 (4)	0.3073 (2)	0.32874 (18)	0.0729 (8)	
H13	0.7257	0.2532	0.3561	0.087*	
C14	0.6243 (4)	0.4007 (2)	0.37115 (16)	0.0589 (7)	
C15	0.5060 (4)	0.4801 (2)	0.33155 (17)	0.0704 (8)	
H15	0.4903	0.5429	0.3613	0.085*	
C16	0.4093 (4)	0.4668 (2)	0.24666 (17)	0.0679 (8)	
H16	0.3289	0.5211	0.2198	0.081*	
Cl1	0.74755 (12)	0.41813 (6)	0.47717 (5)	0.0868 (3)	
N1	0.3387 (3)	0.35391 (18)	0.11479 (13)	0.0642 (6)	

### Atomic displacement parameters (Å^2^)

**Table:**

	*U*^11^	*U*^22^	*U*^33^	*U*^12^	*U*^13^	*U*^23^
C1	0.0509 (15)	0.0545 (15)	0.0543 (15)	−0.0032 (12)	0.0145 (12)	−0.0052 (12)
C2	0.0647 (18)	0.0620 (17)	0.0567 (16)	−0.0099 (13)	0.0161 (13)	−0.0195 (13)
C3	0.0560 (16)	0.0773 (19)	0.0477 (14)	−0.0106 (14)	0.0076 (12)	−0.0068 (14)
C4	0.0553 (16)	0.0606 (16)	0.0514 (15)	−0.0046 (13)	0.0042 (12)	0.0065 (12)
C5	0.0395 (13)	0.0540 (15)	0.0532 (14)	−0.0032 (11)	0.0097 (11)	−0.0038 (11)
C6	0.0386 (13)	0.0526 (14)	0.0451 (13)	−0.0037 (11)	0.0113 (10)	−0.0036 (11)
C7	0.0478 (14)	0.0525 (15)	0.0507 (14)	−0.0031 (12)	0.0097 (11)	−0.0070 (12)
C8	0.102 (2)	0.0502 (16)	0.0739 (19)	0.0005 (15)	0.0191 (17)	−0.0075 (13)
C9	0.098 (2)	0.118 (3)	0.0494 (16)	−0.013 (2)	0.0029 (16)	−0.0095 (17)
C10	0.0644 (18)	0.0555 (16)	0.0653 (17)	0.0012 (13)	0.0087 (13)	−0.0035 (13)
C11	0.0577 (16)	0.0568 (15)	0.0468 (14)	−0.0031 (13)	0.0067 (12)	0.0032 (12)
C12	0.109 (2)	0.0492 (15)	0.0595 (17)	0.0074 (16)	0.0064 (16)	0.0013 (13)
C13	0.091 (2)	0.0588 (17)	0.0618 (17)	0.0151 (16)	−0.0005 (16)	0.0133 (14)
C14	0.0611 (17)	0.0606 (17)	0.0495 (15)	−0.0013 (13)	−0.0004 (12)	0.0066 (12)
C15	0.087 (2)	0.0643 (18)	0.0535 (16)	0.0166 (16)	0.0004 (15)	−0.0049 (13)
C16	0.075 (2)	0.0669 (18)	0.0560 (16)	0.0206 (15)	0.0018 (14)	0.0017 (14)
Cl1	0.1009 (7)	0.0845 (6)	0.0590 (5)	−0.0013 (5)	−0.0189 (4)	0.0027 (4)
N1	0.0795 (16)	0.0600 (14)	0.0491 (12)	−0.0032 (12)	0.0049 (11)	0.0004 (11)

### Geometric parameters (Å, °)

**Table:**

C1—C2	1.386 (3)	C9—H9B	0.9600
C1—C6	1.419 (3)	C9—H9C	0.9600
C1—C8	1.506 (3)	C10—H10A	0.9600
C2—C3	1.380 (4)	C10—H10B	0.9600
C2—H2	0.9300	C10—H10C	0.9600
C3—C4	1.379 (3)	C11—C12	1.378 (4)
C3—C9	1.520 (3)	C11—C16	1.379 (3)
C4—C5	1.391 (3)	C11—N1	1.419 (3)
C4—H4	0.9300	C12—C13	1.382 (4)
C5—C6	1.408 (3)	C12—H12	0.9300
C5—C10	1.513 (3)	C13—C14	1.364 (4)
C6—C7	1.469 (3)	C13—H13	0.9300
C7—N1	1.254 (3)	C14—C15	1.365 (4)
C7—H7	0.9300	C14—Cl1	1.740 (3)
C8—H8A	0.9600	C15—C16	1.388 (4)
C8—H8B	0.9600	C15—H15	0.9300
C8—H8C	0.9600	C16—H16	0.9300
C9—H9A	0.9600		
			
C2—C1—C6	118.4 (2)	H9A—C9—H9B	109.5
C2—C1—C8	118.1 (2)	C3—C9—H9C	109.5
C6—C1—C8	123.5 (2)	H9A—C9—H9C	109.5
C3—C2—C1	123.1 (2)	H9B—C9—H9C	109.5
C3—C2—H2	118.5	C5—C10—H10A	109.5
C1—C2—H2	118.5	C5—C10—H10B	109.5
C4—C3—C2	117.8 (2)	H10A—C10—H10B	109.5
C4—C3—C9	121.3 (3)	C5—C10—H10C	109.5
C2—C3—C9	120.8 (2)	H10A—C10—H10C	109.5
C3—C4—C5	122.2 (2)	H10B—C10—H10C	109.5
C3—C4—H4	118.9	C12—C11—C16	117.8 (2)
C5—C4—H4	118.9	C12—C11—N1	117.5 (2)
C4—C5—C6	119.3 (2)	C16—C11—N1	124.7 (2)
C4—C5—C10	118.3 (2)	C11—C12—C13	121.7 (3)
C6—C5—C10	122.4 (2)	C11—C12—H12	119.1
C5—C6—C1	119.2 (2)	C13—C12—H12	119.1
C5—C6—C7	118.4 (2)	C14—C13—C12	119.2 (2)
C1—C6—C7	122.3 (2)	C14—C13—H13	120.4
N1—C7—C6	125.9 (2)	C12—C13—H13	120.4
N1—C7—H7	117.0	C13—C14—C15	120.7 (2)
C6—C7—H7	117.0	C13—C14—Cl1	119.7 (2)
C1—C8—H8A	109.5	C15—C14—Cl1	119.7 (2)
C1—C8—H8B	109.5	C14—C15—C16	119.7 (3)
H8A—C8—H8B	109.5	C14—C15—H15	120.1
C1—C8—H8C	109.5	C16—C15—H15	120.1
H8A—C8—H8C	109.5	C11—C16—C15	120.9 (3)
H8B—C8—H8C	109.5	C11—C16—H16	119.6
C3—C9—H9A	109.5	C15—C16—H16	119.6
C3—C9—H9B	109.5	C7—N1—C11	119.9 (2)
			
C6—C1—C2—C3	0.0 (4)	C5—C6—C7—N1	169.1 (2)
C8—C1—C2—C3	179.8 (2)	C1—C6—C7—N1	−14.4 (4)
C1—C2—C3—C4	−0.8 (4)	C16—C11—C12—C13	−2.9 (4)
C1—C2—C3—C9	179.4 (2)	N1—C11—C12—C13	178.6 (3)
C2—C3—C4—C5	1.2 (4)	C11—C12—C13—C14	1.8 (5)
C9—C3—C4—C5	−179.0 (2)	C12—C13—C14—C15	0.4 (4)
C3—C4—C5—C6	−0.7 (4)	C12—C13—C14—Cl1	179.8 (2)
C3—C4—C5—C10	−179.8 (2)	C13—C14—C15—C16	−1.3 (4)
C4—C5—C6—C1	−0.2 (3)	Cl1—C14—C15—C16	179.3 (2)
C10—C5—C6—C1	178.9 (2)	C12—C11—C16—C15	2.0 (4)
C4—C5—C6—C7	176.4 (2)	N1—C11—C16—C15	−179.6 (3)
C10—C5—C6—C7	−4.5 (3)	C14—C15—C16—C11	0.1 (5)
C2—C1—C6—C5	0.5 (3)	C6—C7—N1—C11	176.6 (2)
C8—C1—C6—C5	−179.3 (2)	C12—C11—N1—C7	−143.8 (3)
C2—C1—C6—C7	−176.0 (2)	C16—C11—N1—C7	37.8 (4)
C8—C1—C6—C7	4.2 (4)		
